# Production of Water-Soluble Carbohydrates from Aspen Wood Flour with Hydrogen Chloride Gas

**DOI:** 10.1021/acs.iecr.3c01894

**Published:** 2023-10-04

**Authors:** A. Topias Kilpinen, Timo Pääkkönen, Kaarlo Nieminen, Eero Kontturi

**Affiliations:** † Department of Bioproducts and Biosystems, Aalto University, P.O. Box 16300, FI-00076 Aalto, Finland; ‡ Nordic Bioproducts Group Oy, Tietotie 1, 02150 Espoo, Finland

## Abstract

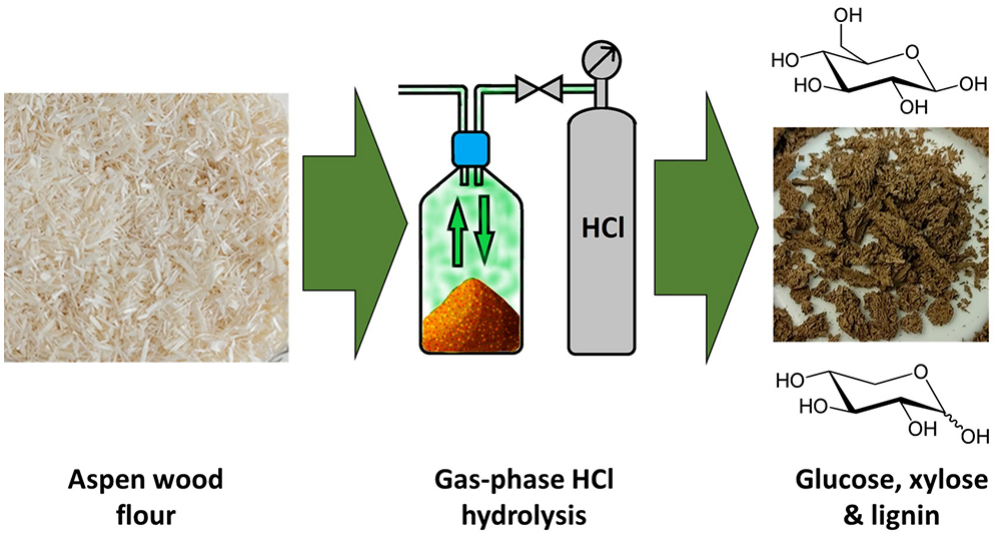

The aim of this study was to optimize the reaction conditions for concentrated acid hydrolysis of aspen wood flour by employing anhydrous hydrogen chloride gas to produce fermentable sugars. Gas hydrolysis with HCl was conducted both with and without temperature control during hydrolysis under a relatively low pressure of 0.1 MPa. Process parameters for HCl gas hydrolysis included the moisture content of aspen wood flour (0.7–50%) and reaction time under pressure (30 min to 24 h). In addition, liquid-phase hydrolysis with concentrated hydrochloric acid was conducted in concentrations of 32–42% and 15 min to 24 h reaction times for comparison with the gas-phase process. The highest yields (>90%) for water-soluble carbohydrates from aspen wood flour were achieved with temperature-controlled gas hydrolysis using 50% moisture content and 2 h total reaction time, which is in line with the previous research and comparable to hydrolysis with concentrated (42%) hydrochloric acid.

## Introduction

Lignocellulose biomass offers a renewable and abundant raw material source, with an estimated annual production of 181.5 billion tons.^1^ It is composed primarily of the biopolymers, cellulose, hemicellulose, and lignin.^2^ From these biopolymers, cellulose and hemicellulose can be depolymerized via hydrolysis to glucose and xylose. These hydrolysis products can then be further processed to different platform chemicals, biofuels, and polymers.^3^

In addition to enzymatic hydrolysis,^4^ acid hydrolysis is seen as the most viable process for hydrolysis of biomass. Acid hydrolysis can be divided into two categories: dilute acid hydrolysis and concentrated acid hydrolysis.^5^ Out of these two hydrolysis processes, the concentrated acid hydrolysis seems interesting from an industrial perspective, as it would enable the complete hydrolysis of crystalline cellulose at ambient temperature with relatively short reaction times and high end-product yields.^6^ Yet utilizing high acid concentrations in the process complicates acid recovery and the purification of desired reaction products, thus rendering the process undesirable for industrial use. It is possible, however, to overcome these problems by employing anhydrous HCl gas for hydrolysis.

Hydrolysis of biomass with anhydrous HCl gas is a gas–solid system and offers several advantages when compared to conventional liquid–solid-based acid systems. In the gas-phase, the HCl is sorbed at ambient conditions into trace amounts of water within the solid biomass, which causes the formation of hydrochloric acid, catalyzing the hydrolysis. The formed mono- and oligosaccharides are retained within the solid phase alongside lignin and other residual components, allowing for their subsequent recovery through a washing step. The benefits of a gas–solid system are the possibility of using nearly fully dry biomass for hydrolysis and the ease of acid separation via evaporation.^7^ In fact, it has been used to convert biomass into oligo- and monosaccharides in various studies and pilot-scale operations since the late 19th century, but it has never enjoyed a wider popularity within the community.^8−11^ Furthermore, systematic comparisons between liquid and gaseous HCl systems for biomass hydrolysis do not exist.

As with other mineral acids, the capacity of HCl to hydrolyze crystalline cellulose is related to the acid concentration in the water phase. When the HCl concentration is below 40%, it can hydrolyze crystalline cellulose only partially. However, when the acid concentration rises above 40%, the crystalline regions of cellulose will break down.^11^ This phenomenon could be explained by comparing a concentrated acid to an ionic liquid. When a certain acid concentration with a mineral acid is reached, there are very few free water molecules left in the system, and it will be composed of only anions, cations, and hydrates. At this point, the acid will start to act similar to an ionic liquid, swelling, decrystallizing, and eventually completely dissolving the crystalline cellulose with parallel hydrolysis to smaller cellulose oligomers and glucose.^12^ And as the dissolution can be conducted even at room temperature, the conversion of cellulose to smaller oligosaccharides proceeds faster than the formation of the degradation products.^13^ Concentrated acid hydrolysis is typically followed by posthydrolysis with dilute acids (0.5–10% or 20–30%) at higher temperatures (70–120 °C) to hydrolyze the remaining cellulose and hemicellulose oligomers to their monomeric form.^14^

In HCl gas hydrolysis, 10% moisture content in biomass should be optimal from the perspective of limiting heat formation while still having enough water in the system for the hydrolysis/decrystallization to take place. However, this 10% optimal moisture content was determined when using relatively high pressures of 0.5–4.2 MPa.^11,15,16^ This is due to the fact that higher pressure during impregnation increases the extent of hydrolysis.^16^ Some of the studies and pilot plants have utilized lower pressures for HCl gas, but in these processes, the gas has mainly been either exposed to wet biomass without pressurizing in a fluidized bed reactor^9,11,17^ or blown through the hydrolysis feedstock in a packed bed reactor.^9^ Yet lower HCl pressures would reduce the burden of recycling and pose more lenient requirements for the instruments. It seems only Antonoplis et al. (1983)^15^ reported hydrolysis of wood flour with a constant 0.1 MPa pressure, but the setup and its results are mentioned only fleetingly aside from a comprehensive treatise on a high-pressure system.

In this study, wood flour from aspen (*Populus tremula*) was hydrolyzed both with concentrated hydrochloric acid and gas-phase HCl by employing the gas hydrolysis reactor used by Pääkkönen et al. (2018).^7^ European aspen (*P. tremula*) and its hybrids are compelling raw material sources for biomass-derived carbohydrates. This is due to their fast growth, high holocellulose content, and their ability to sprout new shoots from roots for quick reforestation.^18^ The aim was to compare liquid- and gas-phase HCl hydrolysis processes and to find optimal parameters to produce fermentable sugars while using a relatively low HCl gas pressure of 0.1 MPa directly on untreated biomass.

## Materials and Methods

### Materials

Aspen (*P. tremula*) wood chips were provided by Avantium NV (The Netherlands). Phenolphthalein (1%) in 50% ethanol, 1 N NaOH, and hydrochloric acid stock solutions (32 and 36.1%) were purchased from VWR. Analytical grade furfural, 5-hydroxymethylfurfural (HMF), and 25% analytical grade sulfuric acid were purchased from Merck. Millipore grade water (resistivity 18.2 MΩ, conductivity 0.8 μS/cm) was used for chromatography analyses. Deionized water was obtained from BIO2 department’s deionized water system with water softener, reverse osmosis, ion exchanger, and UV-light manufactured by Eurowater (conductivity < 1 uS/cm).

### Methods

Carbohydrate composition of oven-dried aspen wood flour was determined according to the analytical method NREL/TP-510-42618.^19^ Sugars were quantified with high-performance anion exchange chromatography with pulsed amperometric detection (HPAEC-PAD) under a Dionex ICS-3000 system (Sunnyvale, CA, USA). Milli-Q water was used as the mobile phase at a flow rate of 0.38 mL/min with a CarboPac PA20 column. Extractives were determined according to the analytical method SCAN-CM 49:03, and ash content was determined according to the analytical method NREL/TP-510-42622.^20^ Sugar composition in hydrolysis filtrates was determined according to the analytical method NREL/TP-510-42623.^21^ Dry matter content of the samples was determined according to the analytical method NREL/TP-510-42621.^22^ Furfural and HMF were determined via high-performance liquid chromatography (HPLC) by using Dionex UltiMate 3000 HPLC (Dionex, Sunnyvale, CA, USA) equipment outfitted with an ultraviolet (UV) detector and Rezex ROA-Organic Acid column (Phenomenex). Sulfuric acid solution (0.0025 mol/L) was used as the eluent at a flow rate of 0.5 mL/min. The column temperature was 55 °C. Furfural and HMF concentrations in the liquid samples were determined by the UV detector at wavelengths of 210 and 280 nm.

### Aspen Carbohydrate Composition

Aspen wood chips were manufactured from debarked aspen logs sourced from Europe. They had been predried at the pilot plant at 105 °C for 24 h in a trayed convection oven. After receiving the wood chips, they were milled into fine wood flour with a Wiley mill M02 through a 1.9 mm screen prior to carbohydrate analysis and hydrolysis. The moisture content of the flour after milling was 0.7%. Carbohydrate composition of aspen wood flour is presented in Table 1.

**Table 1:** Raw Material Aspen Composition

cellulose	xylan	mannose	arabinose	rhamnose	galactan	klason lignin	acid-soluble lignin	extractives	ash	other
45.1%	18.2%	2.5%	0.5%	0.4%	0.6%	19.8%	3.1%	1.7%	0.5%	7.6%

### Acid Hydrolysis of Aspen Wood Flour with Concentrated Aqueous Hydrochloric Acid

Aspen wood flour was hydrolyzed with aqueous hydrochloric acid in concentrations of 32, 36.1, 39, and 42%. Stock hydrochloric acids were first titrated with 1 N NaOH and phenolphthalein to determine the actual acid concentration prior to hydrolysis and concentration with HCl gas. Hydrochloric acids in concentrations of 32 and 36.1% were used as received from VWR for the concentrated acid hydrolysis of aspen wood flour. For the manufacturing of 39 and 42% hydrochloric acid, 36.1% HCl (aq) was concentrated by utilizing the HCl gas reactor.^7^ Acid concentration was assessed via the following protocol. First, 250 mL of 36.1% acid was measured into a 1 L glass reactor bottle using volumetric pipettes, and the weight of the acid was measured. After this, the reactor bottle was placed in an ice bath at a temperature of −1 °C. Once the acid had cooled down, anhydrous HCl gas was absorbed to hydrochloric acid with 0.12 MPa overpressure and mild shaking of the reactor bottle until the desired weight corresponding to a new concentration was reached. The final concentration of the concentrated acid was also checked via titration. The desired weight increase was calculated via the following equation
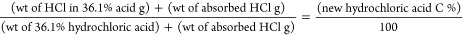
1For the concentrated acid hydrolysis, 2.5 g of 99.3% dry matter content aspen wood flour was measured into nine 250 mL Pyrex bottles. After this, 25 mL of acid in the desired concentration was inserted into each bottle with a volumetric pipet. After acid addition, the bottles were sealed with caps and placed in a rotary shaker running at 200 rpm inside a fume hood at 21 °C. Shaking times for the bottles were 15 min, 30 min, and 1, 2, 3, 4, 6, 8, 16, and 24 h. Once the reaction time had passed, the bottle in question was removed from the shaker and the acid was diluted with 500 g of deionized water. Diluted samples were left to stabilize for 24 h at room temperature. After 24 h, the sample was filtered with a Büchner funnel through 10 μm wire fabric to separate water-soluble oligo- and monosaccharides from residual lignin and insoluble carbohydrates. The filtered hydrolysis residue was then air-dried in a fume hood and measured for dry weight. This dry residue mass was then compared to the calculated dry mass of the hydrolysis starting material for percent weight loss. Residual lignin was analyzed from some sample points via FTIR and NMR. Alterations in the lignin structures were observed, and a comprehensive study focusing on these changes will be published later in another forum. Water-soluble carbohydrates in the hydrolysis filtrates were quantified both directly from concentrated acid hydrolysis filtrate without a posthydrolysis step and from the autoclaved hydrolysis filtrates.

### Acid Hydrolysis of Aspen Wood Flour with Gaseous Hydrogen Chloride

Aspen wood flour with moisture contents (MC) of 1, 5, 10, 15, 20, 30, 40, and 50% was hydrolyzed with anhydrous HCl gas under pressure for 30 min and 2, 6, and 24 h. Concentrated acid hydrolysis with anhydrous HCl gas was conducted in an HCl gas reactor. A pressure of 0.1 MPa was used for the application of HCl gas, and the HCl absorption to aspen wood flour was monitored with a scale. One set of experiments was conducted at room temperature without external cooling or heating and another with temperature control during hydrolysis. Temperature-controlled (TEMP) gas hydrolysis trial points were cooled in an ice water bath for the first 15 min of hydrolysis during the gas application phase at −1 °C and heated by submerging the 1 L glass reactor bottle into 3.7 L of 55 °C water for the last 10 min of the gas hydrolysis. This was done to prevent excess degradation of C5-sugars during the heat formation in the gas application phase and to speed up the hydrolysis reaction during the last 10 min. Between the cooling and heating stages, the gas hydrolysis bottle was kept at room temperature inside the fume hood. For the 30 min reaction times, the bottle was instead placed in a 21 °C water bath for 5 min between cooling and heating to speed up the heating process. The amount of HCl gas applied to the temperature-controlled samples was monitored on a scale to match the sample points without temperature control. After hydrolysis with pressurized HCl gas, the gas was released from the bottle through a neutralization system. The mass change of the system was recorded, and the hydrolysis reaction was stopped. This was done by adding 1000 g of room-temperature deionized water to the sample. Diluted samples were mixed and left to soak for 24 h. Samples were then filtered with a Büchner funnel through 10 μm wire fabric to separate water-soluble oligo- and monosaccharides from residual lignin and insoluble carbohydrates. The filtered hydrolysis residues and hydrolysis filtrates were analyzed by using the same procedures as those with concentrated hydrochloric acid hydrolysis.

### Furanic Degradation Products

Furanic decomposition products were determined from the following hydrolysis filtrates via HPLC calculation of sugar yield from aspen wood flour (see Table 2).

**Table 2:** List of Samples from Which the Furanic Degradation Products were Measured

hydrolysis conditions	reaction times
37% HCl (aq)	15 min, 2 h, 6 and 24 h
42% HCl (aq)	15 min, 2 h, 6 and 24 h
5% MC HCl gas hydrolysis	30 min, 2 h, 6 and 24 h
5% MC HCl gas hydrolysis TEMP	30 min, 2 h, 6 and 24 h
20% MC HCl gas hydrolysis	30 min, 2 h, 6 and 24 h
20% MC HCl gas hydrolysis TEMP	30 min, 2 h, 6 and 24 h
50% MC HCl gas hydrolysis	30 min, 2 h, 6 and 24 h
50% MC HCl gas hydrolysis TEMP	30 min, 2 h, 6 and 24 h

### Calculation of Sugar Yield from Aspen Wood Flour

Sugar yield from aspen wood flour was calculated based on the total available sugar in wood flour according to carbohydrate analysis via the following equation
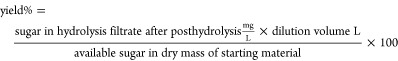
2

### Hydrolysis Modeling

Concentrated acid hydrolysis of aspen wood flour with liquid and gas hydrolysis was modeled from sugar yields in filtrates without posthydrolysis by utilizing a chain scission model with constant scission probability (Table S1 in Supporting Information).

## Results and Discussion

### Hydrolysis of Aspen Wood Flour with Liquid Hydrochloric Acid

**Figure 1:**
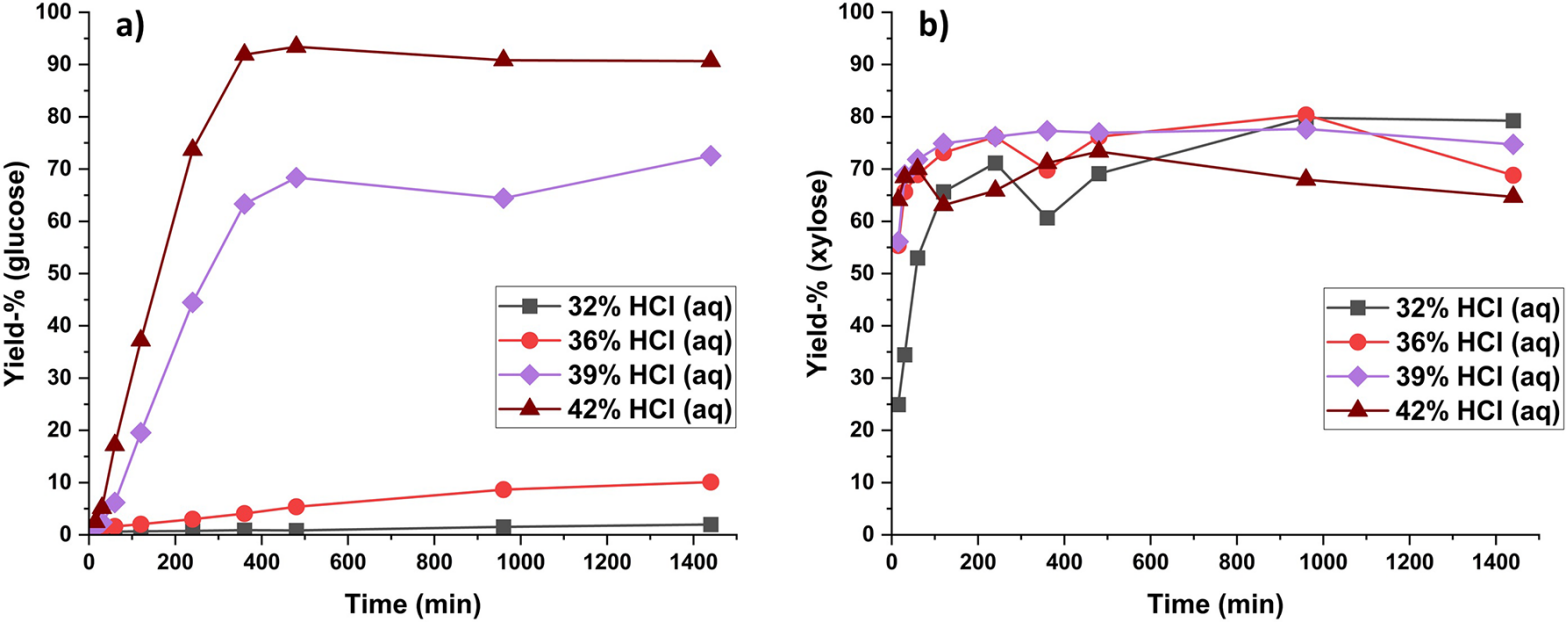
Yield of glucose (a) and xylose (b) (water-soluble carbohydrates) from aspen wood flour on hydrolysis with liquid hydrochloric acid at different acid concentrations over time at 21 °C. Standard deviation for glucose was 5.3 and 1.5 percentage points for xylose.

**Figure 2:**
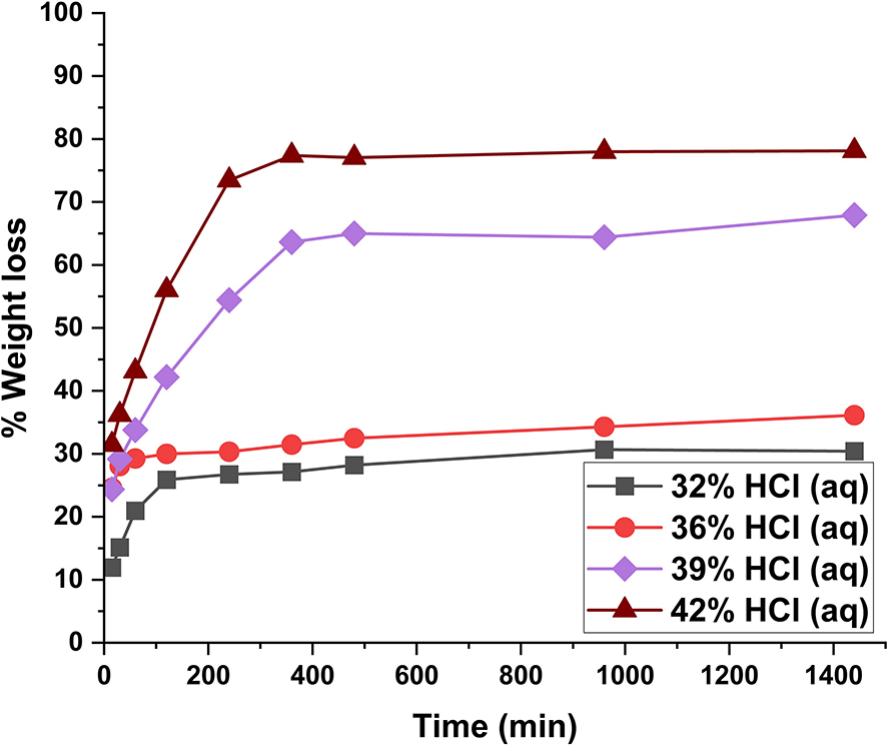
Mass loss expressed as weight loss % of aspen wood flour in hydrolysis with liquid hydrochloric acid at different acid concentrations. Weight loss % with 42% acid is near to the Klason lignin content of aspen wood flour.

Water-soluble carbohydrate yields from hydrolysis with HCl (aq) are listed in Figure 1. It is observable from Figure 1 that with HCl (aq) concentrations of 32 and 36%, mainly the hemicellulose fraction is hydrolyzed (to xylose, Figure 1b), along with some degradation of disordered regions of cellulose (to glucose, Figure 1a). However, the crystalline regions of cellulose remain largely unaffected, with the glucose yield being only 10% even after 24 h hydrolysis in 36% HCl. When the concentration of HCl is increased to 39%, ca. 70% of the cellulose is hydrolyzed to water-soluble oligosaccharides after 6 h and 80% after 24 h. When the HCl concentration is further increased to 42%, the yield of glucose rises to over 90% after 6 h. This indicates that nearly all crystalline cellulose has dissolved and broken down to water-soluble mono- and oligosaccharides during the hydrolysis. This increasing hydrolysis efficiency with acid concentration can also be observed in the weight loss of aspen wood flour (Figure 2). With 42% HCl (aq), the weight loss plateaus close to 80%, which corresponds to the acid-insoluble lignin fraction of aspen wood. This indicates that only lignin remains in the hydrolyzed matrix as nearly all polysaccharides have been hydrolyzed. The results from the hydrolysis with 42% HCl (aq) are comparable with the experiment carried out by Higgins and Ho in 1982, in which they hydrolyzed α-cellulose in 41.7% HCl (aq) at 20 °C temperature, resulting in 90% cellulose weight loss of cellulose after 6 h.^9^

**Figure 3:**
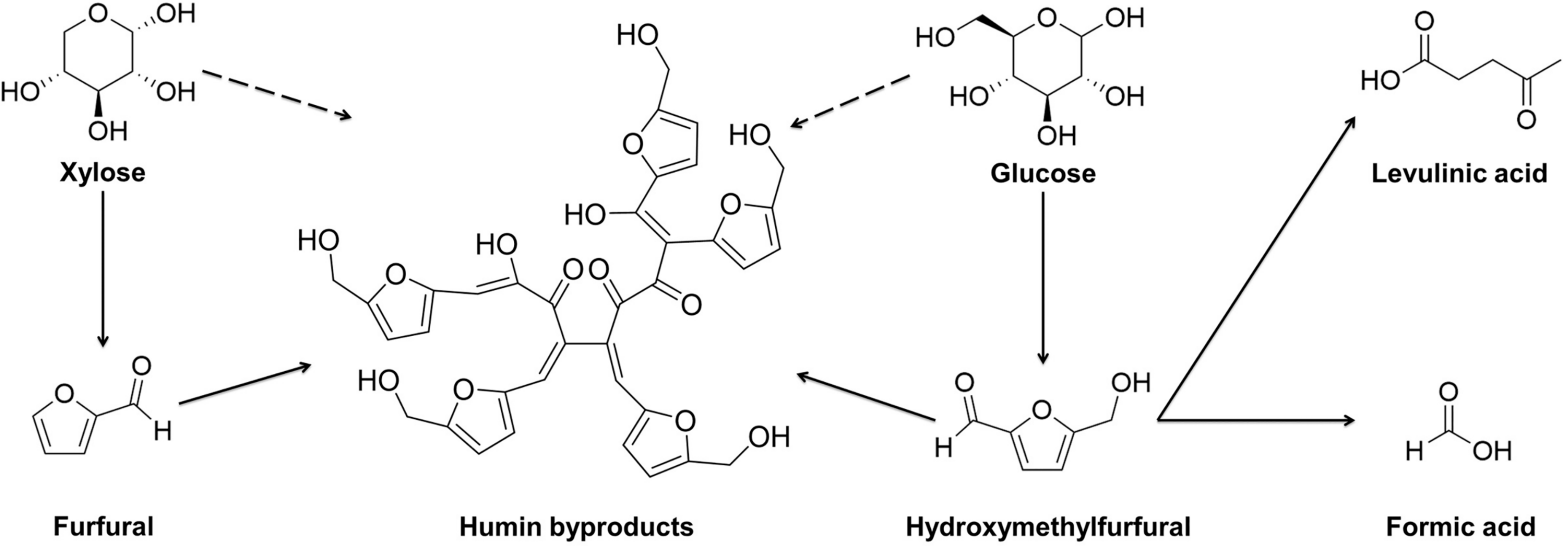
Degradation of glucose and xylose under acidic conditions over time.

From the yield of xylose (Figure 1b), we can see that with all employed acid concentrations the maximum yield of xylose plateaus at ca. 70% after 2 h of hydrolysis. This plateau could potentially be attributed to the fact that, under high acid concentrations, xylose may degrade into furfural and other degradation products like humins as the hydrolysis process advances. This degradation process of xylose and glucose under acidic conditions is illustrated in Figure 3. In acidic conditions, xylose degrades to furfural and glucose degrades to HMF, which in turn can then degrade to levulinic and formic acids. Furfural, HMF, and monosaccharides can also condense into humins, which are heterogeneous carbon-based macromolecules.^23^ However, according to the HPLC results, the amount of furfurals formed during the concentrated acid hydrolysis was quite negligible unless they turned immediately to humins or acids. The highest amount of furans formed during hydrolysis with 42% (HCl aq) after 24 h corresponded to only 4% of the available xylose for furfural and 0.22% of the available glucose for HMF in the sample.

In samples hydrolyzed with HCl (aq), it was observed after dilution that the cellulose fraction had undergone transformation via swelling, dissolution, and regeneration. This phenomenon was most clearly visible in the hydrolysis with 42% hydrochloric acid. After 30 min of hydrolysis, most of the remaining cellulose fraction was decrystallized and dissolved into acid, but after dilution with water, the cellulose regenerated and formed regenerated amorphous cellulose. As the hydrolysis progressed, the dissolved cellulose chains were gradually hydrolyzed to smaller water-soluble oligosaccharides and monomers. Eventually, after 8 h of hydrolysis most of the crystalline cellulose along with the hemicellulose fractions were broken down to water-soluble carbohydrates, leaving behind residual lignin (Figure S1a, Supporting Information).

### Hydrolysis of Aspen Wood Flour with HCl Gas

**Figure 4:**
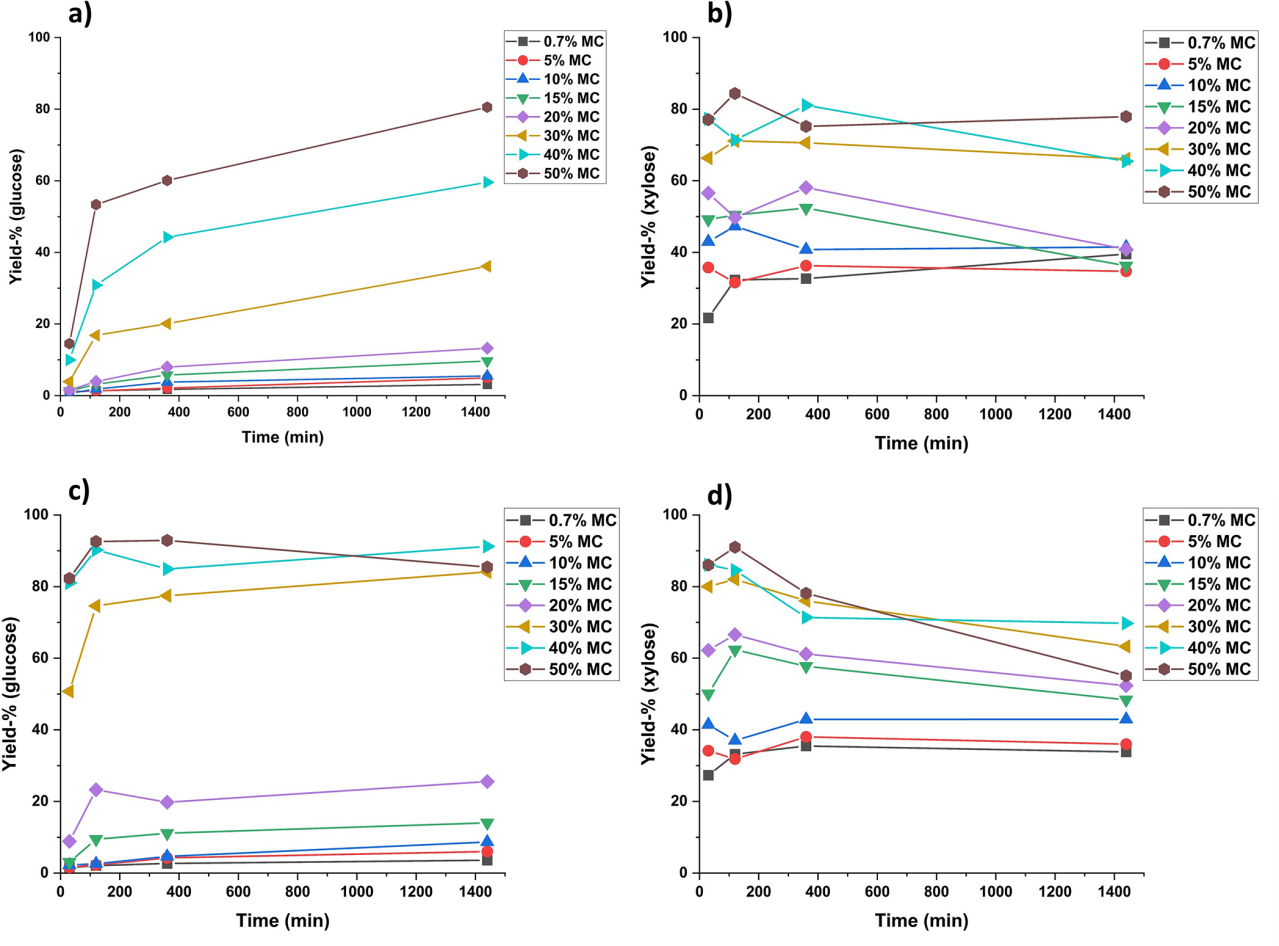
Yield of glucose from aspen wood flour in gas hydrolysis without (a) and with temperature control (c) and for xylose in gas hydrolysis without (b) and with temperature control (d). Standard deviation for glucose was 3.8 and 4.0 percentage points for xylose.

**Figure 5:**
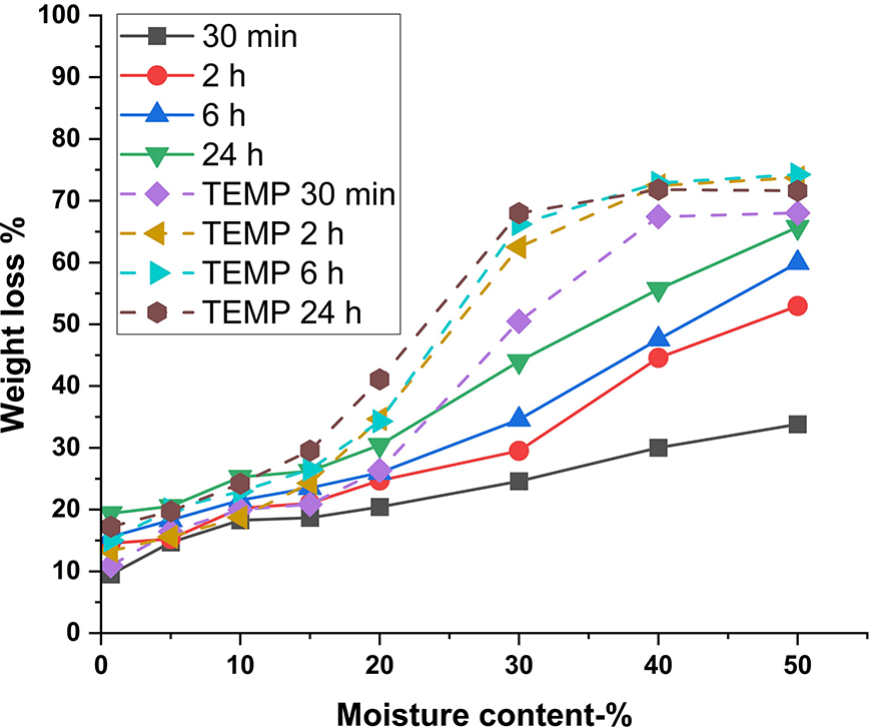
Weight loss % of aspen wood flour in hydrolysis with gaseous HCl hydrolysis. Weight loss % with temperature-controlled samples in 2, 6, and 24 h reaction times in moisture content range of 40–50% are similar to those in 42% liquid HCl hydrolysis after 4 h. TEMP refers to samples with temperature control.

When the polysaccharide hydrolysis is investigated as a function of time in different moisture contents (Figure 4), a dramatic increase in the yield of hydrolysis by HCl (g) is observed between samples with and without temperature control during hydrolysis. This is similar to the results of Higgins and Ho (1982).^9^ In the gas hydrolysis without temperature control, the yield of glucose increases with reaction time and moisture content, but the xylose yield starts to decrease with increased reaction time due to sugar degradation. However, the glucose yield starts to rise over 80% only with longer reaction times of 24 h and a moisture content of 50%. With temperature control, the hydrolysis efficiency is significantly improved. It is possible to gain over 80% yields for both glucose and xylose already after 30 min of hydrolysis in moisture contents of 40% and 50%. Longer reaction times than 30 min increase the glucose yields to over 90%. This increased hydrolysis efficiency with temperature control is also observable from the percentual weight loss during the hydrolysis (Figure 5). Overall, the yields from gas hydrolysis are comparable to the results by Antonoplis et al. (1983),^15^ where up to 85% of the potential glucose and 78% of total sugars were recovered after 5–12 h of hydrolysis under 0.1 MPa pressure.

With temperature-controlled gas hydrolysis, the glucose yield starts to rise significantly at moisture contents of 20% and above. This could be explained by two factors. First of all, wood itself is a poor conductor of heat,^24^ so more water in the wood flour should increase the heat transfer during the heating phase. Second, it was observed that in the sample points with moisture content of 15% and higher, the wood flour would turn into a sticky intermediate product at the early stage of the hydrolysis. This intermediate product would then cover the inside layer of the reactor vessel during rotational mixing in the gas application phase. This in turn would lead to even more intense heat transfer during the heating, when compared to dryer samples where the wood flour would form a self-insulating pile at the bottom of the reactor vessel (Figure S1b, Supporting Information). There is also the issue of the acid concentration in the wood flour during hydrolysis. The absorbed HCl amount during hydrolysis with each moisture content was calculated from residual weight after releasing the extra gas from the reaction bottle and comparing it to the starting weight of water in wood flour. The results are presented in Table 3.

**Table 3:** Average HCl Concentration in Gas Hydrolysis Samples

moisture content %	average HCl concentration in samples based on remaining HCl (%)
0.7	6.1
5	23.1
10	35.1
15	42.2
20	45.7
30	49.2
40	52.4
50	54.6

From the table, we can see that the concentration of acid in the samples exceeds the required 42% HCl concentration in samples with 15% moisture content and higher. The lower acid concentration in the low moisture content wood flours at 0.1 MPa pressure could explain their low glucose yields, even with temperature-controlled gas hydrolysis. However, these calculations are not as accurate with smaller moisture contents, as the water that would trap the HCl molecules after hydrolysis might have been consumed during the hydrolysis reaction. This is also the case with higher moisture content samples, although it would mean that the HCl concentration has been actually higher than calculated. Nevertheless, it is apparent that at least with samples with 15% moisture content and above, it is possible to reach over 42% HCl concentrations in wood flour by employing 0.1 MPa pressure.

Low glucose yields in 0.7–15% moisture contents could be explained by the inability of HCl to access water inside the fiber and the cellulose structure. At lower moisture contents below the fiber saturation point, water exists either as clusters of bound water between fibrils or as tightly bound water on the cellulose fibril surfaces.^25^ With the relatively low pressure used in this study (0.1 MPa), the HCl gas might not be able to dissociate in the tightly bound layer of water on the surface of fibrils in low moisture content samples. This would also explain why it is still possible to gain high glucose yields with just ∼10% moisture content when using higher HCl gas pressures (4.2 MPa), as in this case, the HCl molecules would have enough kinetic energy to break the bond between water and cellulose fibrils.

In addition, although the sugar yields are over 90%, this indicates just how much of the available sugar from the wood flour has been converted to the water-soluble form during concentrated acid hydrolysis. The actual sugar yields after posthydrolysis are ∼5% lower for glucose and ∼15% lower for xylose due to sugar degradation at 120 °C. This could be mitigated by using a lower temperature and a shorter posthydrolysis time in the autoclave. For example, according to Yoon et al.,^6^ the temperature should be kept at 100 °C to reduce glucose degradation to furans and further on to organic acids. Moreover, some of the carbohydrates linked to lignin are not recovered because the residue is not posthydrolyzed along with the filtrate, thus lowering the yield of water-soluble carbohydrates. Such behavior is similar to the hydrolysis with liquid acid. In consequence, the sugar yield could presumably be increased by also posthydrolyzing the residue.

### Furan Formation in Hydrolysis with Liquid and Gaseous HCl

**Figure 6:**
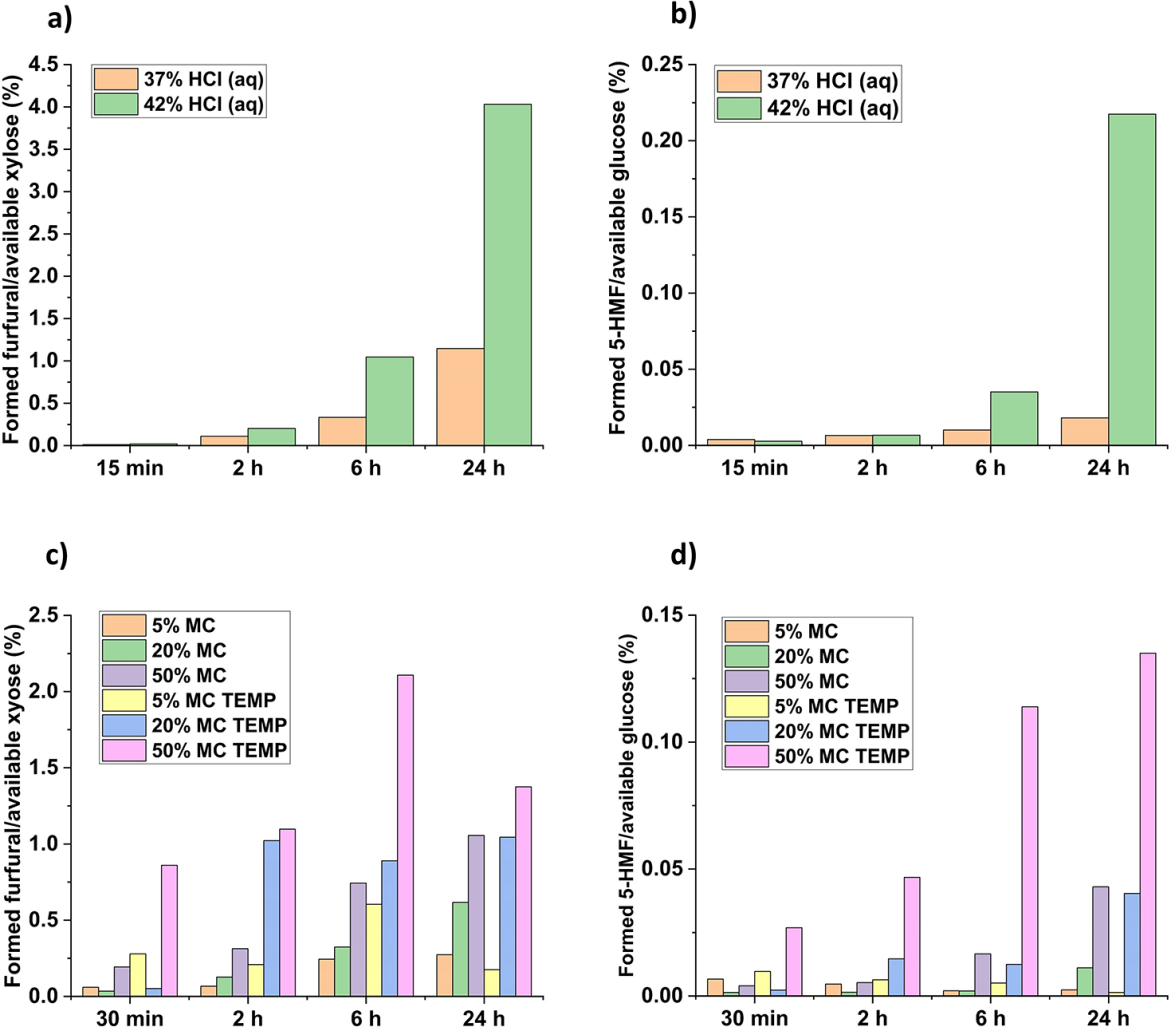
Furan formation during concentrated acid hydrolysis measured with HPLC. Furfural (a) and HMF (b) formation with HCl (aq) and furfural (c) and HMF (d) formation with gaseous HCl. TEMP refers to samples with temperature control. Formation is expressed as the percentage of furfural formed from available xylose in the sample and as the percentage of HMF formed from available glucose in sample.

HMF and furfural formations, as determined by HPLC in the filtrate, are presented in Figure 6. In hydrolysis with HCl (aq), the furan formation increases with the reaction time, as shown in Figure 6a,b.

Furan formation is more intense in hydrolysis with 42% HCl (aq) than in 37% HCl (aq) due to a higher acid concentration. However, even with a 24 h reaction time using 42% HCl (aq), only 4% of the available xylose in the sample turned to furfural and only 0.2% of the available glucose turned to HMF. This is due to the dehydration condition: furfural generation is preferred over HMF generation when the pH is below 2.^26^ In the same sample, 65% of the available xylose and 92% of the available glucose were converted to water-soluble carbohydrates during concentrated acid hydrolysis.

Gas hydrolysis samples without temperature control exhibit similar behavior with increasing moisture content and reaction time leading to an increase in furan formation, as shown in Figure 6c,d. However, in samples with temperature control, the furan formation plateaus and even decreases with 24 h reaction times, especially in case of furfural. This behavior could be explained by humin formation. Heating at the end of temperature-controlled gas hydrolysis is probably increasing the humin formation, which can be observed as the darkening of the sample during hydrolysis heating. The overall darkening of the hydrolysis residue is also more extensive with higher moisture content samples and is clearly visible in the difference between samples with 0.7% MC and 20% MC after six h temperature-controlled gas hydrolysis (Figure S1c, Supporting Information). Hydrolysis samples with HCl (aq) also exhibited similar darkening behavior during hydrolysis, indicating that those samples also exhibit humin formation. As with the hydrolysis with HCl (aq), xylose is more susceptible to degradation than glucose in HCl gas hydrolysis. For example, the highest amount of furfural formed during the gas hydrolysis was 22 mg in 6 h temperature-controlled gas hydrolysis (Figure 6c), which corresponds to only 2% of the total available xylose. In the same sample point, only 0.14% of the available glucose was degraded to HMF (Figure 6d).

Overall, the amount of furans formed during concentrated acid hydrolysis is quite negligible under the utilized reaction conditions. However, the furans are turning eventually into humins, which are more complex to quantify, and thus, the actual sugar degradation is probably higher. In addition, HMF is degraded to formic acid and levulinic acid as the hydrolysis progresses.

### Hydrolysis Modeling

For the hydrolysis with HCl (aq), the portion of cellulose undergoing scission rises with the acid concentration, and no further degradation is observed (Figure S2a, Supporting Information). The portion of xylan undergoing scission also rises with acid concentration, but the xylose starts to degrade at acid concentrations of 37–42% over time (Figure S2b, Supporting Information).

For the HCl gas hydrolysis, the portion of cellulose and xylan undergoing scission depends on the moisture content in the samples. The rate of hydrolysis for cellulose is higher with a temperature-controlled sample (Figure S3a,b, Supporting Information). As with the HCl (aq), no further degradation is observed with glucose (Figures S3a,b, Supporting Information), but xylose is subjected to degradation over time as the moisture content increases (Figure S3c,d, Supporting Information). However, the degradation is more moderate with a temperature-controlled sample.

Only a restricted number of insights can be interpreted from the calculated scission rate constants and xylose degradation rates (Table S2, Supporting Information). In general, increasing chain scission and xylose degradation with increasing acid concentration or moisture content is observable. There is also indication of further degradation for xylose monomers but not for glucose monomers. However, the parameter estimates are not very accurate due to scarcity of data points especially with gas hydrolysis. In addition, the rapid initial xylose increase obstructs the scission rate estimation for gas hydrolysis as there are no sample points between 0 and 30 min reaction time.

### Comparison between Gas and Liquid Hydrolysis and with Earlier Research

When comparing the results between these hydrolysis pathways, we can make a couple of observations. First of all, the highest glucose yield from gas hydrolysis without temperature control was lower (80% with 50% MC and 24 h reaction time) than that from hydrolysis with 42% HCl (aq) (94%). However, temperature-controlled gas hydrolysis produced glucose yields comparable to hydrolysis with 42% HCl (aq) due to an increased rate of hydrolysis during the heating stage. Second, in temperature-controlled gas hydrolysis with 30 min and 2 h reaction times, the xylose yields at moisture contents of 40 and 50% are slightly higher (∼90%) than the xylose yields from hydrolysis with HCl (aq) in all used concentrations (∼60 to 80%.) This might be caused by cooling, preventing initial degradation, and heating increasing the reaction rate for hydrolysis of hemicellulose at the end. In future studies, it would be interesting to repeat the hydrolysis series with HCl (aq) but at different reaction temperatures or with heating at the end of the hydrolysis. In any case, it was possible to gain high sugar yields with both hydrolysis pathways investigated in this study without any pretreatment of wood flour.

Results from this study were also compared to some of the earlier studies that have utilized HCl gas hydrolysis for biomass. These studies are presented in Table 4. As can be seen from Table 4, the yields achieved in this study are comparable to the previous studies utilizing gaseous HCl, although direct comparison is difficult due to the high variety in process parameters. The closest reaction conditions were utilized by Antonoplis et al.^15^ with 0.1 MPa pressure and 15 mL glass ampules, but the more detailed setup and results were not specified as ampule runs were part of preliminary tests for larger scale tests with a fluidized bed reactor. It is also noteworthy that the sugar yields achieved with HCl gas hydrolysis and with concentrated HCl (aq) in this and the previous studies are much higher than the sugar yields achieved with dilute acid hydrolysis. In dilute acid hydrolysis, the yields of recovered sugars are in the range of 50% due to monosaccharide degradation in higher process temperatures.^28^

**Table 4:** Some of the Earlier Studies with Hydrolysis of Biomass with Gaseous HCl

feedstock	moisture content	reactor	pressure/flow rate	temperature	reaction time	yield	references
α-cellulose, newspaper, wheat straw and wheat hulls	50%	packed bed reactor/Liebig condenser	0.014 MPa and gas flow rate of 2.7 L per minute	20–50 °C	5–180 min	94–96% of the cellulose hydrolyzed within 35 min	(9)
pine sawdust	58.10%	10.5 L column type reactor	150 (L/min)	18 °C at inlet, 40–70 °C at the outlet	1 h	91.50%	(27)
2 mm Wiley-milled *Populus tristis*	not specified	15 mL glass ampules	0.1 MPa	temperature reduction with liquid nitrogen to condensate the gas. Passive warming to room temperature for hydrolysis	5–12 h	up to 85% of the potential glucose and 78% of the total sugars	(15)
2 mm Wiley-milled *P. tristis*	50%	stainless-steel bombs equipped with glass liners	0.34–0.55 MPa	with precooling to 0 °C and saturation with gaseous HCI at 0.1 MPa before pressurization temperatures did not exceed 60°C	5 h	with precooling 80% conversion of glucose was achieved	(15)
2 mm Wiley-milled *P. tristis*	6.40%	pressurized fluidized bed reactor	0.55–1.37 MPa	not specified/27°C	1 h	80% glucose 90% xylose	(15)

Nevertheless, despite being proven multiple times that it is possible to gain high sugar yields from biomass with relatively mild reaction conditions, there are still challenges with scaling the process up to the industrial level. The most prominent challenges are the low heat conductivity of the hydrolyzed wood particles/biomass and the formation of sticky intermediate reaction product during gas hydrolysis. Until this challenge can be solved, concentrated acid hydrolysis employing a liquid–solid system would be a more viable option to HCl gas hydrolysis from the industrial perspective.

## Conclusions

It was possible to gain high yields of water-soluble carbohydrates from aspen wood flour with both liquid and gaseous HCl hydrolysis. In addition, monosaccharide degradation to furans during concentrated acid hydrolysis was minimal. Temperature-controlled gas hydrolysis produced high yields for both xylose and glucose without a prehydrolysis step, even with a relatively low pressure of 0.1 MPa. The highest water-soluble carbohydrate yields from available xylan and glucan were 91 and 92%, respectively. These yields were achieved with temperature-controlled gas hydrolysis at 50% moisture content using a 2 h reaction time. The results are consistent with previous research employing anhydrous HCl gas for hydrolysis.

## Supporting Information Available

Equations used for modeling, modeling data, and photographs of hydrolysis residues to visualize the hydrolyzed samples (PDF)
