# Supplementary material for: Production of Water-Soluble Carbohydrates from Aspen Wood Flour with Hydrogen Chloride Gas
Source: Ind Eng Chem Res. 2023 Oct 4;62(41):16922–30. doi: 10.1021/acs.iecr.3c01894 (PMC10588446; doi:10.1021/acs.iecr.3c01894)
Supplement: Supplementary file 1 — ie3c01894_si_001.pdf [file ie3c01894_si_001.pdf]

Supporting Information

Production of water-soluble carbohydrates from aspen wood flour with hydrogen chloride gas

A. Topias Kilpinen [a], Timo Pääkkönen [a,b], Kaarlo Nieminen [a] and Eero Kontturi [a]*

[a] Department of Bioproducts and Biosystems, Aalto University, P.O. Box 16300, FI-00076 Aalto, Finland

[b] Nordic Bioproducts Group Oy, Tietotie 1, 02150 Espoo, Finland

* Corresponding author: Eero Kontturi, eero.kontturi@aalto.fi

Table of contents

Photographs of hydrolysis residue	S2
Chain scission model	S3
Fits for the chain scission model	S4
Rate constants acquired from modeling	S6

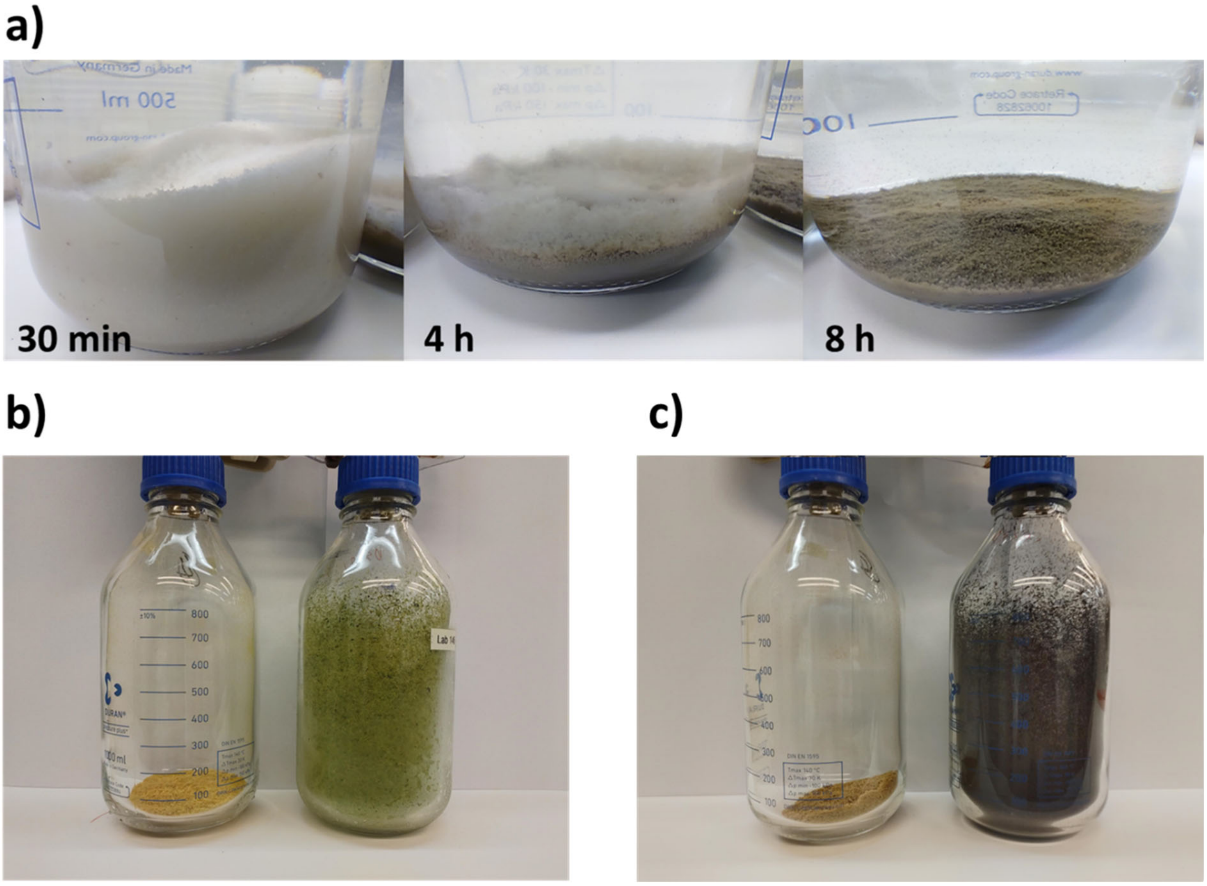

Figure S1. Photographs of hydrolysis residue after dilution in 30-minute, 4 h and 8 h hydrolysis with 42 % hydrochloric acid. After 8 hours of hydrolysis residue consists mainly from Klason lignin. a) Samples with moisture content of 0.7% (left) and 20% moisture content (right) after 20 minutes of hydrolysis in temperature controlled HCl gas hydrolysis. b) Samples with moisture content of 0.7% (left) and 20% moisture content (right) after 6 hours of hydrolysis in temperature controlled HCl gas hydrolysis prior to final heating. c)

Table S1. Chain scission model with constant scission probability.

$\frac{dx}{dt} = -\alpha\, x$	(S1)	Scission velocity proportional to remaining bonds
$\frac{dy}{dt} = 2\alpha \cdot x - 2\alpha \cdot y$	(S2)	Increase in end groups also proportional to remaining bonds excluding already existing end groups
$\frac{dz}{dt} = \alpha \cdot y$	(S3)	Increase in monomers proportional to number of end groups (No monomer degradation)
$\frac{dz}{dt} = \alpha \cdot y - \beta \cdot z$	(S4)	If degradation of monomers occurs, subtracted term proportional to number of monomers

Number of bonds – x
Number of end groups – y
Number of monomers – z

Rate constants – α, β

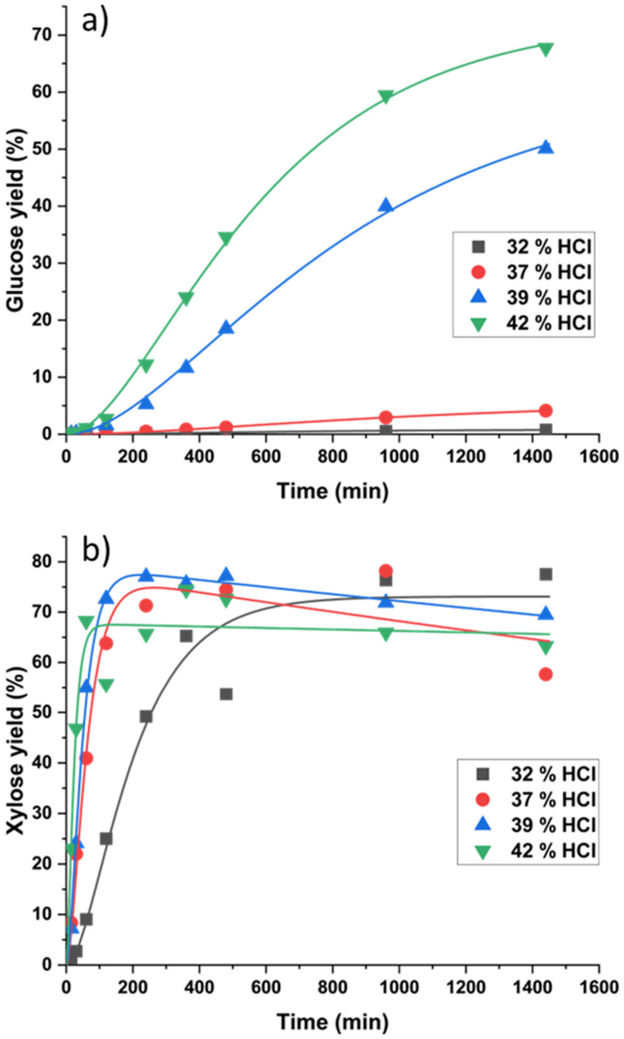

Figure S2. Chain scission model for cellulose (a) and xylan (b) in hydrolysis with HCl (aq).

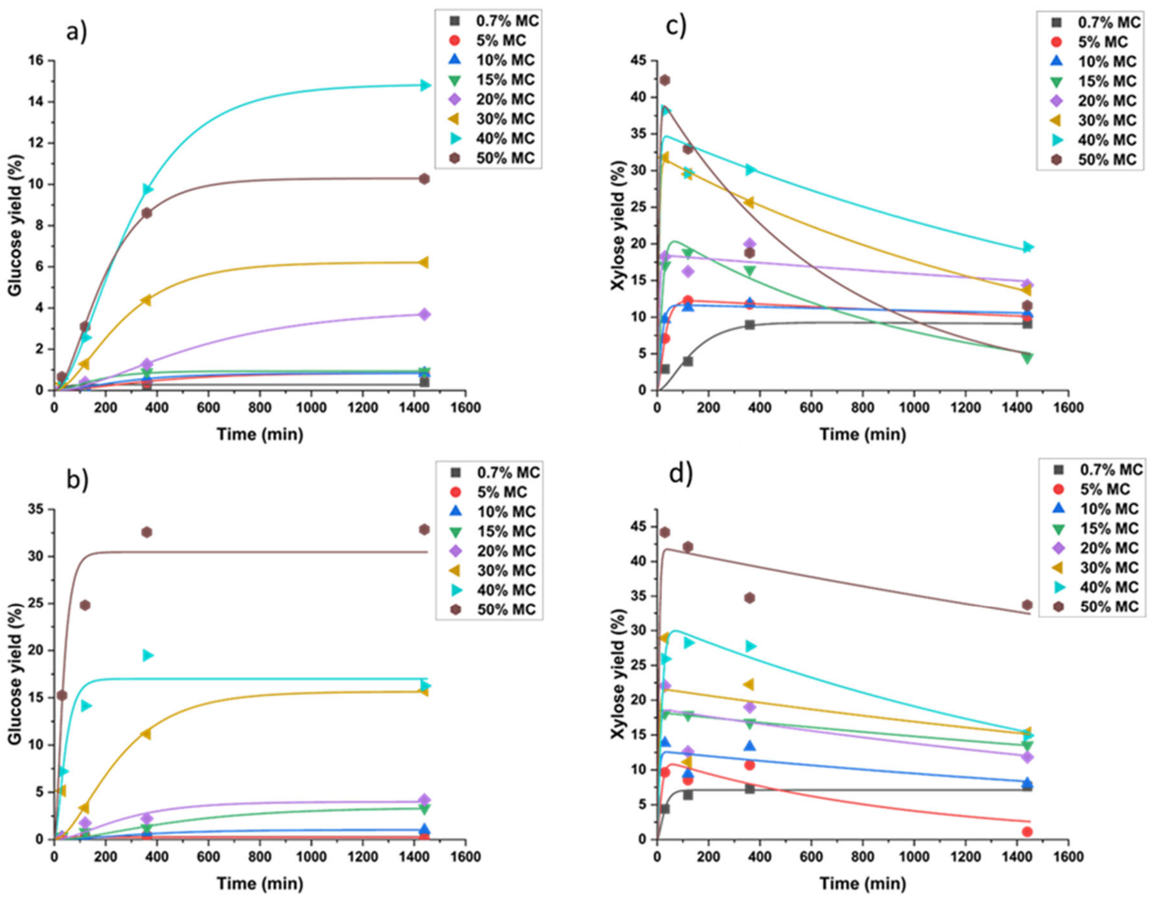

Figure S3. Chain scission model for cellulose in gas hydrolysis without (a) and with temperature-control (b) and for xylan in gas hydrolysis without (c) and with temperature-control (d).

Table S2.

a) Cellulose scission rate constant and scissioning portion in hydrolysis with HCl (aq).

HCl concentration	Scission rate constant	Scissioning portion
%	min^{-1}	%
32	0.0020	0.83
37	0.0013	5.68
39	0.0016	61.95
42	0.0024	73.17

b) Xylan scission rate constant, xylose degradation rate and scissioning portion in hydrolysis with HCl (aq).

HCl concentratio n	Scission rate constant	Xylose degradatio n rate	Scissionin g portion
%	min^{-1}	min^{-1}	%
32	0.007	0.0	73.1
37	0.022	0.000134	77.3
39	0.028	0.000095	79.0
42	0.060	0.000022	67.7

c) Cellulose scission rate constant and scissioning portion in hydrolysis with gaseous HCl without temperature-control.

Moisture content	Scission rate constant	Scissioning portion
%	min^{-1}	%
0.7	0.0385	0.3
5	0.0035	0.9
10	0.0048	0.8
15	0.0081	0.9
20	0.0024	3.9
30	0.0051	6.2
40	0.0046	14.9
50	0.0068	10.3

d) Xylan scission rate constant, xylose degradation rate and scissioning portion in hydrolysis with gaseous HCl.

Moisture content	Scission rate constant	Xylose degradation rate	Scissioning portion
%	min^{-1}	min^{-1}	%
0.7	0.010	2.4E-05	9.4
5	0.047	1.5E-04	12.4
10	0.080	7.3E-05	11.7
15	0.075	1.0E-03	21.6
20	0.178	1.5E-04	18.5
30	0.200	6.0E-04	31.9
40	0.200	4.3E-04	35.2
50	0.200	1.5E-03	40.3

e) Cellulose scission rate constant and scissioning portion in hydrolysis with gaseous HCl with temperature-control.

Moisture content	Scission rate constant	Scissioning portion
%	min^{-1}	%
0.7	0.0488	0.3
5	0.1935	0.2
10	0.0040	1.0
15	0.0029	3.4
20	0.0050	4.0
30	0.0054	15.6
40	0.0319	17.0
50	0.0386	30.5

f) Xylan scission rate constant, xylose degradation rate and scissioning portion in hydrolysis with gaseous HCl.

Moisture content	Scission rate constant	Xylose degradatio n rate	Scissionin g portion
%	min^{-1}	min^{-1}	%
0.7	0.051	0.00E+00	7.1
5	0.087	1.04E-03	11.4
10	0.200	2.93E-04	12.7
15	0.200	2.10E-04	18.2
20	0.200	3.10E-04	18.8
30	0.200	2.50E-04	21.7
40	0.084	4.89E-04	30.9
50	0.200	1.81E-04	42.0
